# Distribution of Nidogen in the Murine Eye and Ocular Phenotype of the Nidogen-1 Knockout Mouse

**DOI:** 10.5402/2012/378641

**Published:** 2012-08-08

**Authors:** Christian Albrecht May

**Affiliations:** Department of Anatomy, Faculty of Medicine Carl Gustav Carus, TU Dresden, 01307 Dresden, Germany

## Abstract

Distribution and lack of nidogen-1, part of numerous basement membranes, were studied in the mouse eye. For that purpose, eyes of C57BL/6 and nidogen-1 knockout mice were stained immunohistochemically for nidogen-1, and intraocular pressure measurements and light- and electron microscopy were used to study the nidogen-1 knockout animals. In normal mice, nidogen-1 was present in many basement membranes, but showed irregularities underneath the corneal epithelium, in Bruch's membrane and in the iris. Homozygous knockout of nidogen-1 in the mouse showed only mild pathological changes. In the anterior eye segment, small interruptions were noted in the nonpigmented ciliary epithelium without further consequences. In the posterior eye segment, interruptions of the inner limiting membrane led to small retinal ectopias and subsequent changes in the optic nerve. In summary, the knockout of nidogen-1 showed mild but significant morphological changes pointing to the importance of this protein which can in part, but not completely; be replaced by nidogen-2.

## 1. Introduction

Basement membranes form the natural supporting structure upon which cells migrate, proliferate, and differentiate. They contain a tissue-specific composition of extracellular matrix components, containing collagen type IV, laminin, heparan sulfate proteoglycans, and nidogen. In mammals the nidogen family consists of two members, nidogen 1 and 2. Both isoforms bind to a wide spectrum of BM-associated proteins, and it has been proposed that they act as connecting elements between the laminin and collagen IV networks [1–4].

Nidogen-1-deficient animals show only mild phenotypes [5–7]; most BMs are ultrastructurally normal, and there is little change in cellular or tissue morphology. The homozygous knockout animals are generally healthy, have a normal lifespan, and are fertile. Specific settings reveal mild neurological abnormalities in these animals [5, 8]. Nidogen-2-deficient animals show no primary phenotype, but are more sensitive to pathologies like hypertension [9] and cancer [10–12]. Double mutants lacking both isoforms die shortly after birth with abnormalities directly related to defects in BM assembly [13, 14].

In the eye, nidogen-1 is described to be present in numerous basement membranes. Studies in the mouse are, however, restricted to the cornea and retina [15, 16]. The aim of this study was to investigate the distribution of nidogen in the normal adult murine eye and to describe the phenotype after nidogen-1 knockout. In our primary hypothesis, we suspected a link between basement membrane changes and ocular hypertension which, however, could not be verified.

## 2. Material and Methods

For the distribution of nidogen-1 in the eye, five three-month-old C57BL/6 mice were used. To study the phenotype of nidogen-1 knockout mice, ten animals were kindly provided from the colony described previously [6, 13, 14]. In this colony, exon 3 of the NID1 gene was deleted. Genetic testing revealed that two of these animals had a wild-type genotype, four were heterozygous expressing nidogen-1 to a lower level, and four were homozygous, showing a complete knockout of nidogen-1. All animals were raised and kept in accordance to the federal rules of animal care.

### 2.1. Intraocular Pressure (IOP) Measurements

IOP was measured immediately prior to sacrification of the animals as described previously [17]. In brief, the animals were deeply anesthetized with an intramuscular injection of 20 IU of a 5% ketamine hydrochloride/xylazine solution. The head was fixed on a board and the eye punctured with a 33G needle, connected to a pressure measuring system. The needle remained for two to five minutes in the anterior chamber of the eye with a constant recording of the pressure.

### 2.2. Immunofluorescence

Sagittal and tangential 14 *μ*m thick frozen cryostat sections through the mouse eye were mounted on poly-L-lysine covered glass slides. Incubation was performed overnight at room temperature using a monoclonal rat anti entactin/nidogen 1 antibody (MAB1946; Chemicon International Inc., Temecula, CA, USA) diluted 1 : 200. After rinsing in phosphate buffered saline pH 7.4, a mouse-adsorbed biotinylated goat-anti-rat Ig antibody (Pharmingen, San Diego, CA, USA) was added at the same concentration for one hour. The staining was visualized with a Cy3-conjugated Streptavidin complex (Jackson Immuno Research Laboratories Inc., West Grove, PA, USA), diluted 1 : 1000. The slides were mounted with glycerine jelly and viewed under an Aristoplan fluorescence microscope (Leitz, Wetzlar, Germany).

### 2.3. Light and Transmission Electron Microscopy

Small specimens of the anterior and posterior eye segment and of the optic nerve were immersion fixed in a solution containing 2.5% glutaraldehyde, 2.5% paraformaldehyde, and 0.05% picric acid for several days. The specimens were then rinsed in cacodylate buffer (pH 7.2), incubated in OsO_4_ for 2 hours, dehydrated and embedded in Epon.

Semithin sections of 1 *μ*m were stained with toluidine blue and examined by light microscopy. Ultrathin sections were stained with uranyl acetate and lead citrate and viewed with a Zeiss electron microscope (Zeiss, Oberkochen, Germany).

## 3. Results

### 3.1. Distribution of Nidogen-1 in the Normal Mouse Eye

**Figure 1:**
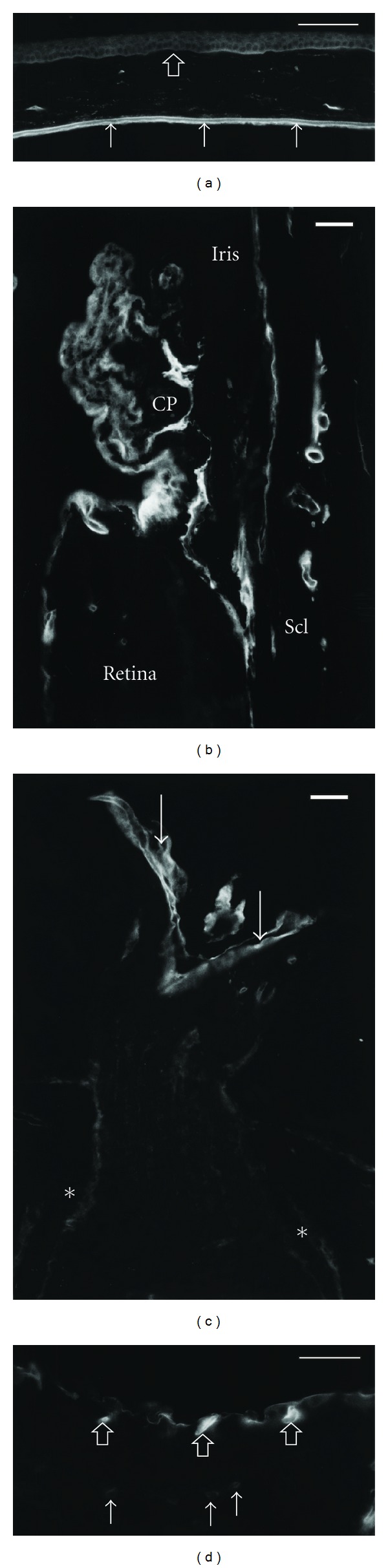
Micrographs of a C57BL/6 mouse eye stained for nidogen-1. Scale bar equals 50 *μ*m. (a) In the cornea, intense staining is seen on both sides of Descement's membrane (arrows). Staining of the corneal epithelium basal membrane is inconsistent (open arrow). (b) In the anterior eye segment, staining is seen at the epithelial cells of the ciliary processes (CP) and around scleral vessels (Scl). (c) At the optic nerve, intense staining is present in the inner limiting membrane (arrows). Staining is also seen towards the border of the pia mater (asterisks). (d) In the retina, the inner limiting membrane and the inner retinal vessels (open arrows) show intense staining, while the outer capillaries (arrows) show less intense staining for nidogen-1.

In the anterior eye segment, nidogen-1 immunoreactivity was seen in the epithelial cells lining the ciliary body, in the trabecular meshwork, and at both sides of Descement's membrane right next to the endothelial cells (Figures 1(a) and 1(b)). No consistent staining could be observed in the basal membrane of the corneal epithelium and within the iris.

In the posterior eye segment, immunoreactivity was seen in the inner limiting membrane of the retina, at the border line of the optic nerve towards the pia mater, and in the basal membrane of larger vessels in the perioptic tissue, sclera, and choroid (Figure 1(c)). Bruch's membrane showed an inconsistent staining. Within the retina, the outer capillaries showed less intense staining than the vessels in the nerve fibre layer (Figure 1(d)).

**Figure 2:**
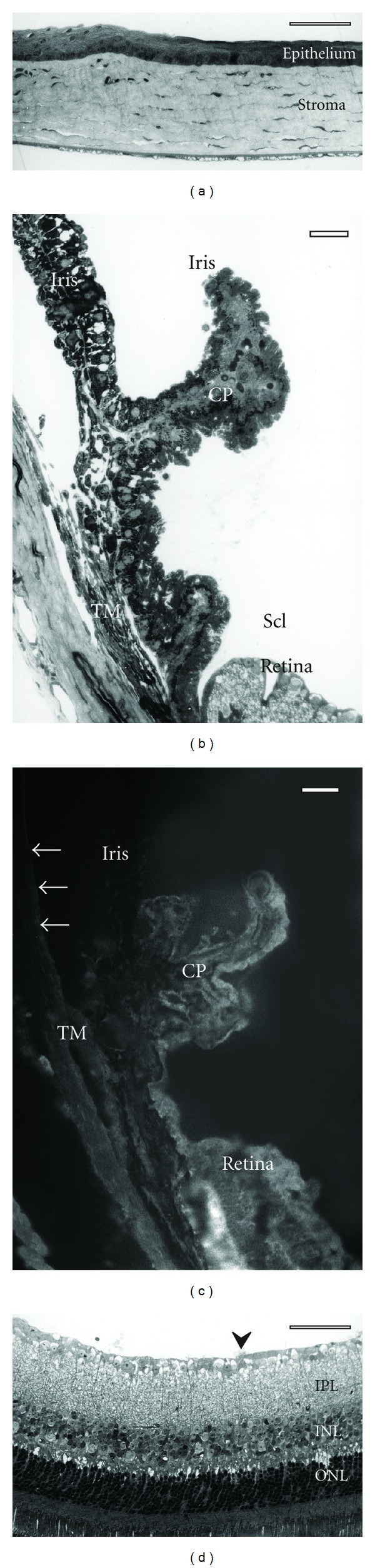
Light microscopic images of a homozygous nidogen-1 knockout mouse. Scale bar equals 50 *μ*m. The morphology of the cornea (a) and ciliary body/iris (b) is not altered. There is no staining for nidogen-1 (c) Descement's membrane (arrows), ciliary processes (CP), and trabecular meshwork (TM). In the retina (d), small protrusions of neuronal tissue can be seen (arrowhead); the retina shows otherwise no morphological changes. IPL: inner plexiform layer, INL: inner nuclear layer, ONL: outer nuclear layer.

Eyes of the heterozygous nidogen-1 knockout mice revealed the same staining pattern as normal controls. There was also no difference regarding the staining intensity. Homozygous nidogen-1 knockout mice showed no staining indicating true knockout and sensitivity of the antibody for nidogen-1 (Figure 2(c)).

### 3.2. Phenotype of Nidogen-1 Knockout Mouse Eye

Only minimal changes were observed in the anterior eye segment of homozygous nidogen-1 knockout mice. The basal lamina of the nonpigmented epithelium of the ciliary body showed small interruptions (data not shown). The contacting epithelial cells showed no morphological alterations. All other tissues were completely normal (trabecular meshwork, cornea, iris, ciliary muscle; Figures 2(a) and 2(b)). The intraocular pressure was not altered (C57BL/6 mice: 11.5, 12, 12.5 cmH_2_O; wild-type litter mates: 9.5, 10, 11.5 and 12.5 cmH_2_O; heterozygous nidogen-1 knockout: 8.5, 9, 14 cmH_2_O; homozygous nidogen-1 knockout: 14, 14, 11, 14 cmH_2_O).

**Figure 3:**
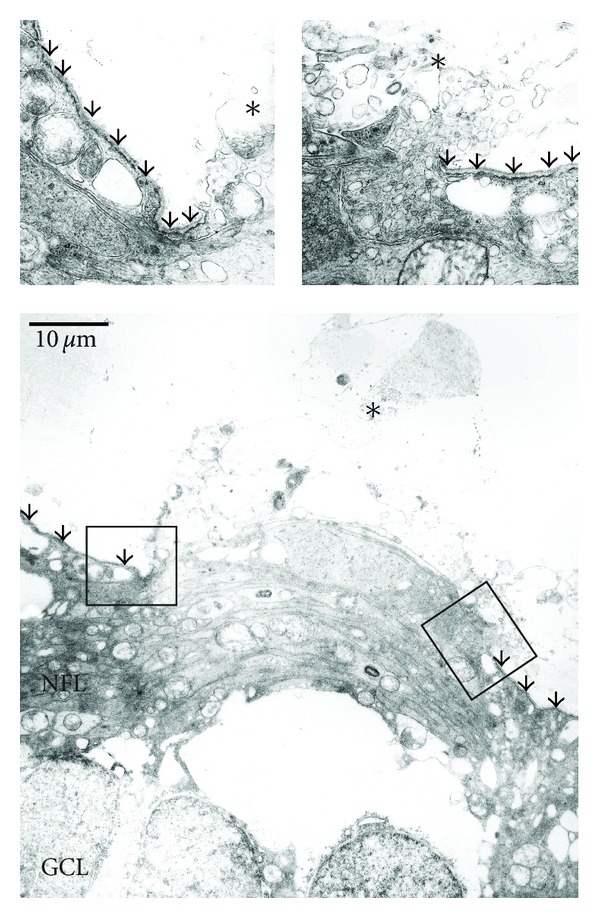
Electron microscopic view of a whole in the inner limiting membrane of a homozygous nidogen-1 knockout mouse. Both edges are shown in higher magnification. The inner limiting membrane is marked with arrows. Note the protruding neural tissue (asterisks). GCL: ganglion cell layer. NFL: nerve fiber layer.

**Figure 4:**
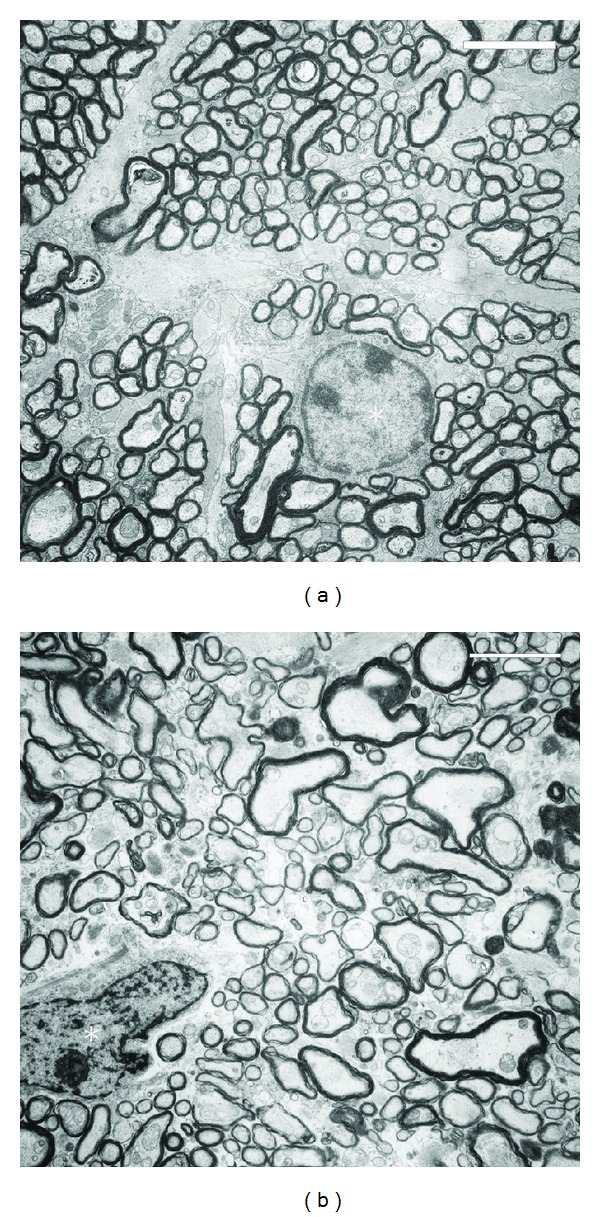
Electron microscopic views of an optic nerve cross section of a heterozygous (a) and a homozygous (b) nidogen-1 knockout mouse (scale bar equals 10 *μ*m). In the heterozygous mouse, the nerve fibres are regular arranged and the astrocytes in a quiescent stage (asterisk). In the homozygous mouse, the nerve fibres are irregular arranged and the astrocytes appear activated (asterisk).

In the posterior eye segment, obvious changes were restricted to the inner limiting membrane of the retina. At places, the basal membrane was completely lacking forming small holes. These areas were more often found in the central region around the optic nerve. Neural tissue prolapsed through these holes into the vitreous cavity (Figures 2(d) and (3)). This tissue contained mostly of optic nerve fibers, occasionally also optic ganglion cells were seen displaced in this prolapsed tissue. Although no significant loss of nerve fibres was seen in optic nerve cross sections (estimated number of optic nerve fibres heterozygous knock-out: 95000, homozygous knockout: 92000; area of optic nerve cross section heterozygous knockout: 190376 *μ*m^2^, homozygous knock-out: 185165 *μ*m^2^), an increase of degenerating nerve fibres was detected in the homozygous knockout mice versus the heterozygous animals and the controls (Figure 4).

The remaining retinal tissue, Bruch's membrane, and the choroid appeared completely normal.

## 4. Discussion

### 4.1. Anterior Eye Segment

The distribution of nidogen-1 in the C57BL/6 mouse cornea confirmed the findings of Balb/c mice [15] and of young human donors [18, 19]. The inconsistent staining in the corneal epithelial basement membrane showed no local characteristics as known for other basement membrane components [20]. Similar to human, bovine, and newt eyes [18, 21, 22] nidogen-1 is present in the murine trabecular meshwork, pronounced toward the outflow channels (Schlemm's canal). For the first time, we describe the presence of nidogen-1 in both basement membranes of the ciliary epithelium.

Since nidogen-1 is present in all tissues related to aqueous humour turnover we carefully studied possible changes in homozygous nidogen-1 knockout mice. To our surprise, there was no severe morphological alteration other than small holes in the basement membrane of the nonpigmented ciliary epithelium. These changes did not affect the underlying epithelial cells. The trabecular meshwork appeared completely unchanged. The functional integrity was confirmed by measuring normal levels of intraocular pressure.

The changes in the lens capsule present in nidogen-1 knockout mice [5] could not be investigated in these animals due to the direct intraocular pressure measurements.

### 4.2. Posterior Eye Segment

Within the retina we extended the finding described in 1 and 12 months old C57BL/6 mice [16] to the fact that nidogen-1 is already present in the inner limiting membrane of 3 months old animals. Therefore, nidogen-1 is not only present in older animals but already in younger adults. Furthermore, we observed an inconsistent staining of Bruch's membrane. There is no visual data on nidogen distribution in the human retina but it is mentioned as basement-membrane-like distributed in the tables of some publications [23, 24].

The inner limiting membrane disruptions found in homozygous nidogen-1 knockout mice were highly specific and present in all animals studied. They were not present in nidogen-2 knockout animals (own unpublished results). The phenomenon of “retinal ectopias” was only recently described in a mouse model of muscle-eye-brain disease [25]. Therefore, knockout of POMGnT1 leads to a thinning of the inner limiting membrane with frequent breaks. Similar changes are described in Large^myd^ mice [26] and after knockout of *β*2 laminin [27]. Interestingly, no literature exists about the described phenomenon of retinal ectopia outside the mouse.

Concerning nidogen, there is no human disease directly related to the lack of nidogen-1. Although antibodies against nidogen were reported in Chagas disease and American cutaneous leishmaniasis [28], the ocular pathology described in these conditions [29, 30] differs markedly from that described in this paper.

Since only the inner limiting membrane was affected, it is tempting to speculate that specific mechanical forces might stress this basement membrane and therefore depend highly on the presence of nidogen. Among these factors, eye movement in general might play a role and, concerning dimensions of the mouse eye, the passive movements of the lens pulling the thin vitreous layer. The latter may also account for the fact that this phenomenon has never been described in animals with a different lens-vitreous ratio.

## References

[1] Aumailley M, Battaglia C, Mayer U, Nischt R, Timpl R, Fox JW (1993). Nidogen mediates the formation of ternary complexes of basement membrane components. *Kidney International*.

[2] Fox JW, Mayer U, Nischt R (1991). Recombinant nidogen consists of three globular domains and mediates binding of laminin to collagen type IV. *EMBO Journal*.

[3] Kimura N, Toyoshima T, Kojima T, Shimane M (1998). Entactin-2: a new member of basement membrane protein with high homology to entactin/nidogen. *Experimental Cell Research*.

[4] Kohfeldt E, Sasaki T, Göhring W, Timpl R (1998). Nidogen-2: a new basement membrane protein with diverse binding properties. *Journal of Molecular Biology*.

[5] Dong L, Chen Y, Lewis M (2002). Neurologic defects and selective disruption of basement membranes in mice lacking entactin-1/nidogen-1. *Laboratory Investigation*.

[6] Murshed M, Smyth N, Miosge N (2000). The absence of nidogen 1 does not affect murine basement membrane formation. *Molecular and Cellular Biology*.

[7] Schymeinsky J, Nedbal S, Miosge N (2002). Gene structure and functional analysis of the mouse nidogen-2 gene: nidogen-2 is not essential for basement membrane formation in mice. *Molecular and Cellular Biology*.

[8] Vasudevan A, Ho MS, Weiergräber M (2010). Basement membrane protein nidogen-1 shapes hippocampal synaptic plasticity and excitability. *Hippocampus*.

[9] Amann K, Haas CS, Zeiler GA (2009). Lack of nidogen-2 increases blood pressure, glomerular and tubulointerstitial damage in DOCA-salt hypertension. *European Journal of Clinical Investigation*.

[10] Kuk C, Gunawardana CG, Soosaipillai A (2010). Nidogen-2: a new serum biomarker for ovarian cancer. *Clinical Biochemistry*.

[11] Cheng ZX, Huang XH, Wang Q, Chen JS, Zhang LJ, Chen XL (2012). Clinical significance of decreased nidogen-2 expression in the tumor tissue and serum of patients with hepatocellular carcinoma. *Journal of Surgical Oncology*.

[12] Mokkapati S, Bechtel M, Reibetanz M, Miosge N, Nischt R (2012). Absence of the basement membrane component nidogen 2, but not of nidogen 1, results in increased lung metastasis in mice. *Journal of Histochemistry & Cytochemistry*.

[13] Bader BL, Smyth N, Nedbal S (2005). Compound genetic ablation of nidogen 1 and 2 causes basement membrane defects and perinatal lethality in mice. *Molecular and Cellular Biology*.

[14] Böse K, Nischt R, Page A, Bader BL, Paulsson M, Smyth N (2006). Loss of nidogen-1 and -2 results in syndactyly and changes in limb development. *The Journal of Biological Chemistry*.

[15] Schittny JC, Timpl R, Engel J (1988). High resolution immunoelectron microscopic localization of functional domains of laminin, nidogen, and heparan sulfate proteoglycan in epithelial basement membrane of mouse cornea reveals different topological orientations. *Journal of Cell Biology*.

[16] Kunze A, Abari E, Semkova I, Paulsson M, Hartmann U (2010). Deposition of nidogens and other basement membrane proteins in the young and aging mouse retina. *Ophthalmic Research*.

[17] Dyka FM, May CA, Enz R (2004). Metabotropic glutamate receptors are differentially regulated under elevated intraocular pressure. *Journal of Neurochemistry*.

[18] Katz A, Fish AJ, Pe’er J, Frucht-Pery J, Ron N, Vlodavsky I (1994). Entactin/nidogen: synthesis by bovine corneal endothelial cells and distribution in the human cornea. *Investigative Ophthalmology and Visual Science*.

[19] Kabosova A, Azar DT, Bannikov GA (2007). Compositional differences between infant and adult human corneal basement membranes. *Investigative Ophthalmology and Visual Science*.

[20] Ljubimov AV, Burgeson RE, Butkowski RJ, Michael AF, Sun TT, Kenney MC (1995). Human corneal basement membrane heterogeneity: topographical differences in the expression of type IV collagen and laminin isoforms. *Laboratory Investigation*.

[21] Dietlein TS, Jacobi PC, Paulsson M, Smyth N, Krieglstein GK (1998). Laminin heterogeneity around Schlemm’s canal in normal humans and glaucoma patients. *Ophthalmic Research*.

[22] Ortiz JR, Vigny M, Courtois Y, Jeanny JC (1992). Immunocytochemical study of extracellular matrix components during lens and neural retina regeneration in the adult newt. *Experimental Eye Research*.

[23] Ljubimov AV, Burgeson RE, Butkowski RJ (1996). Basement membrane abnormalities in human eyes with diabetic retinopathy. *Journal of Histochemistry & Cytochemistry*.

[24] Fukuchi T, Ueda J, Abe H, Sawaguchi S (2001). Cell adhesion glycoproteins in the human lamina cribrosa. *Japanese Journal of Ophthalmology*.

[25] Hu H, Candiello J, Zhang P, Ball SL, Cameron DA, Halfter W (2010). Retinal ectopias and mechanically weakened basement membrane in a mouse model of muscle-eye-brain (MEB) disease congenital muscular dystrophy. *Molecular Vision*.

[26] Lee Y, Kameya S, Cox GA (2005). Ocular abnormalities in Large(myd) and Large(vls) mice, spontaneous models for muscle, eye, and brain diseases. *Molecular and Cellular Neuroscience*.

[27] Pinzón-Duarte G, Daly G, Li YN, Koch M, Brunken WJ (2010). Defective formation of the inner limiting membrane in laminin *β*2- and *β*3-null mice produces retinal dysplasia. *Investigative Ophthalmology and Visual Science*.

[28] Avila JL, Rojas M, Velazquez-Avila G, von der Mark H, Timpl R (1986). Antibodies to basement membrane protein nidogen in Chagas’ disease and American cutaneous leishmaniasis. *Journal of Clinical Microbiology*.

[29] Fröhlich SJ, Miño de Kaspar H, Perán R (1998). Eye involvement in Chagas disease (American trypanosomiasis). 1996/1997 studies in Paraguay. *Ophthalmologe*.

[30] Matsumoto SC, Labovsky V, Roncoroni M (2006). Retinal dysfunction in patients with chronic Chagas’ disease is associated to anti-Trypanosoma cruzi antibodies that cross-react with rhodopsin. *The FASEB Journal*.

